# Bayesian Modelling of Induced Responses and Neuronal Rhythms

**DOI:** 10.1007/s10548-016-0526-y

**Published:** 2016-10-07

**Authors:** Dimitris A. Pinotsis, Roman Loonis, Andre M. Bastos, Earl K. Miller, Karl J. Friston

**Affiliations:** 1 The Picower Institute for Learning & Memory and Department of Brain and Cognitive Sciences, Massachusetts Institute of Technology, Cambridge, MA 02139 USA; 2 The Wellcome Trust Centre for Neuroimaging, University College London, London, WC1N 3BG UK

**Keywords:** Dynamic causal modelling, Intersubject variability, Connectivity, Microelectrodes, Laminar responses, Compartmental models, Hierarchical Bayesian models

## Abstract

Neural rhythms or oscillations are ubiquitous in neuroimaging data. These spectral responses have been linked to several cognitive processes; including working memory, attention, perceptual binding and neuronal coordination. In this paper, we show how Bayesian methods can be used to finesse the ill-posed problem of reconstructing—and explaining—oscillatory responses. We offer an overview of recent developments in this field, focusing on (i) the use of MEG data and Empirical Bayes to build hierarchical models for group analyses—and the identification of important sources of inter-subject variability and (ii) the construction of novel dynamic causal models of intralaminar recordings to explain layer-specific activity. We hope to show that electrophysiological measurements contain much more spatial information than is often thought: on the one hand, the dynamic causal modelling of non-invasive (low spatial resolution) electrophysiology can afford sub-millimetre (hyper-acute) resolution that is limited only by the (spatial) complexity of the underlying (dynamic causal) forward model. On the other hand, invasive microelectrode recordings (that penetrate different cortical layers) can reveal laminar-specific responses and elucidate hierarchical message passing and information processing within and between cortical regions at a macroscopic scale. In short, the careful and biophysically grounded modelling of sparse data enables one to characterise the neuronal architectures generating oscillations in a remarkable detail.

## Introduction

Neural rhythms have been associated with a variety of cognitive functions; including working memory (Pesaran et al. 2002; Siegel et al. 2009), visual attention (Buschman and Miller 2007; Fries 2009; Kornblith et al. 2015; Womelsdorf et al. 2006), cortical representations (Buzsáki and Chrobak 1995; Schoffelen et al. 2005), feature binding (Tallon-Baudry et al. 1996) and information propagation in feedforward/feedback directions in cortical hierarchies (Bastos et al. 2012; Friston et al. 2015). Oscillatory activity is also thought to be the signature of aberrant neuronal processing in psychiatric diseases (Uhlhaas and Singer 2012), such as autism (Dickinson et al. 2015) or schizophrenia (Gonzalez-Burgos and Lewis 2008) and can be used to disclose mechanisms underlying intersubject variability (Pinotsis et al. 2013). Gamma band responses in particular, have been shown to reflect various input attributes, like the size of visual objects (Pinotsis et al. to appeara; Perry et al. 2013), luminance (Swettenham et al. 2013) and contrast (Pinotsis et al. 2014; Ray and Maunsell 2010; Roberts et al. 2013). Here, we consider powerful tools from Bayesian inference to illustrate the wealth of information about brain function that neural rhythms and electrophysiological responses afford. In this setting, Bayesian deconvolution and empirical Bayes are used to finesse the ill-posed problem of reconstructing and explaining electromagnetic sources and oscillatory responses. In this setting, neural activity is described by probability densities parameterized by physiological or anatomical (lead field) parameters and hyper-parameters that embody assumptions about random effects. This description provides a generative model of how underlying signals are caused, which can be used to optimise the model—and its (physiological) parameters. This approach calls on a combination of forward and backward modelling that involves simulating predicted responses (using biologically plausible anatomical models) and Bayesian inversion to estimate cortical structure and function. We will showcase two applications of Variational Bayes to extract information from neuroimaging data, see also (Pinotsis and Friston 2014a): First, we will illustrate the richness of non-invasive recordings by reviewing recent studies that use parametric empirical Bayes (PEB) to characterise intersubject variability—in cortical function—using non-invasive electrophysiology. Second, we will preview a new study that uses laminar data and Bayesian model comparison to analyse oscillatory recordings obtained from the prefrontal cortex during a delayed saccade task. These complementary examples show how biologically informed modelling of electrophysiological measurements can, on the one hand, allow questions about microcircuitry to be answered using macroscopic (non-invasive) data, while on the other hand microscopic (invasive) data can be used to inform hypotheses about neuronal interactions at a macroscopic scale.

## Hierarchical Bayesian Models and the Analysis of Neuroimaging Data

In this section, we summarize some theoretical results and show how (i) parametric empirical Bayes (PEB) can be used to quantify group effects in multi-subject studies—by optimizing hierarchical Bayesian models and (ii) how Bayesian model comparison allows us to reconcile formally distinct (compartmental and mean field) models and construct DCMs of laminar probe data.

The first application provides a fresh perspective on the use of non-invasive electrophysiology: M/EEG are often thought to have excellent temporal but limited spatial resolution, see e.g., (Lütkenhöner 2003). However, in previous work we have shown how variational Bayesian inference can be used as a mathematical microscope to yield non-invasive estimates of cortical anatomy, structure and function, see e.g., (Pinotsis et al. 2013). At the same time, M/EEG responses can illuminate the neurobiological mechanisms underpinning neural rhythms (Friston et al. 2015; Pinotsis et al. 2014). Here, we present further results that illustrate the rich spatial information in non-invasive data and argue that this richness is (only) bounded by the models used to explain empirical data—and the experimental design used to elicit those state (e.g., experimental artefacts, number and location of the sensors, length of recording sessions etc.), see also (Troebinger et al. 2014). Exploiting this rich spatial information rests upon formulating appropriate hierarchical Bayesian models that generate predictions of neural activity and group effects (Friston et al. 2016). A general form of these models accommodates both within and between subject effects. For example:

1 $$\begin{aligned} y_{i} &= \varGamma_{i} (\theta^{(1)} ) + \varepsilon_{i}^{(1)} \\ \theta^{(1)} &= \varGamma (\theta^{(2)} ) + \varepsilon^{(2)} \\ \theta^{(2)} &= \eta + \varepsilon^{(3)} \\ \end{aligned}$$

where $y_{i}$ is a matrix of *i*-th subject responses, $\varGamma_{i} (\theta^{(1)} )$ represents the (differential equation or dynamic causal) model that generates these responses with parameters $\theta^{(1)}$, $\varGamma (\theta^{(2)} )$ is the between subject (second level) model that describes intersubject variability in the parameters of the first level model. The second level maps second to first level parameters (e.g., group means to subject-specific parameters), where $\varepsilon^{(i)}$ represent random effects at each level (e.g., intersubject variability and observation noise). Below, we combine these second level models with models of brain activity that make predictions about the dynamics of coupled excitatory and inhibitory populations. In these applications, $\varGamma_{i} (\theta^{(1)} )$ captures biophysical (see e.g. (Deco et al. 2008; Pinotsis and Friston 2014a) behaviours that are caused by key architectures and (synaptic) connectivity parameters of interest. Bayesian procedures allow us to identify the form of hierarchical models and estimate their (hidden) parameters using observed responses and Variational Bayesian inference (Friston et al. 2007, 2008).

In the context of non-invasive electrophysiology, the hierarchical model (2) poses the difficult inversion problem of finding neural source estimates in the context of intersubject variability. This involves (i) partitioning the covariance of observed data into observation error and components that can be explained in terms of neuronal responses, that themselves entail components due to second level (between-subject) level variability; (ii) exploiting differential equation models to provide anatomical and physiological constraints on the explanation for first level (within subject) responses—usually in terms of (synaptic) connectivity estimates. The second level covariance components specify whether the parameters of the dynamical model at the first level are random or fixed effects, while dynamical models provide predictions of the dynamics at source and sensor space, which depend upon cortical anatomy and physiology.

In summary, hierarchical or empirical Bayesian modelling of the sort implied by Eq. (2) allows us to perform efficient source reconstruction and obtain connectivity estimates by replacing phenomenological constraints (e.g., based on autoregressive modelling and temporal smoothness considerations) by spatiotemporal constraints based on models of neuronal activity. This can be thought as an alternative to autoregressive models, which model statistical dependencies among measured signals—as opposed to the neuronal processes generating measurements. In dynamic causal modelling, one uses a forward or generative model of distributed processing to estimate the (coupling) parameters of that model. Inference then proceeds assuming nonlinear within-subject effects and linear between subject effects. This allows one to distinguish among competing hypotheses about the mechanisms and architectures generating the data and the nature of group effects in multiple subject studies (Friston et al. 2015, 2016; Pinotsis et al.; to appear).

Crucially, the use of Bayesian model reduction (BMR) allows one to reduce the computational burden of inverting PEB models from multiple subjects and enables an efficient scoring and averaging of large sets of (nested) models (Friston and Penny 2011). Bayesian model reduction entails the estimation of a posterior density over hidden model parameters for a reduced model (defined in terms of a prior density) using just the posterior density estimated from a full model (with a complete set of parameters). We can express the generative model in Eq. 1 in terms of a likelihood model and the implicit (empirical) priors:

2 $$\begin{aligned} \ln p(y,\theta^{(1)} ,\theta^{(2)} |m) &= \sum\nolimits_{i} {\ln p(|\theta^{(1)} )} + \ln p(\theta^{(1)} |\theta^{(2)} ) + \ln p(\theta^{(2)} |m) \\ p\left( {y_{i} |\theta^{(1)} ,m} \right) &= {\mathcal{N}}(\varGamma_{i} (\theta^{(1)} ),\varSigma_{i} (\theta^{(1)} )) \\ p\left( {\theta^{(1)} |\theta^{(2)} ,m} \right) &= {\mathcal{N}}(\varGamma (\theta^{(2)} ),\varSigma (\theta^{(2)} )) \\ p\left( {\theta^{(2)} |m} \right) &= {\mathcal{N}}(\eta ,\varSigma ) \\ \end{aligned}$$

Here, $y$ denotes the data obtained from all subjects (indexed by *i*) and the generative model $\varGamma_{i}$ is a function of model parameters at the first or within-subject level: $\theta^{(1)}$. These parameterize the connectivity architecture mediating responses, the observation function $\varphi \subset \theta^{(1)}$ and the spectra of the inputs and channel noise, $\{ \alpha_{n} ,\alpha_{u} ,\beta_{n} ,\beta_{u} \} \subset \theta^{(1)}$. Gaussian assumptions about sampling errors $\varepsilon^{(1)}$ provide the likelihood model at the first (within-subject) level: $p(y_{i} |\theta^{(1)} )$. To explain intersubject variability this model is supplemented with a mapping from group means to subject-specific estimates: $\theta^{(1)} = (X \otimes I)\theta^{(2)} + \varepsilon^{(2)}$, where $\varepsilon^{(2)}$ are random effects (at the between–subject level) and *X* is a design matrix containing between-subject explanatory variables. Below, using Bayesian model reduction, we adjudicate among competing hypotheses about the intrinsic connections that show intersubject variability. Effectively, this involves comparing the evidence for random effects models with and without (combinations of) between subject effects on (combinations) of connectivity parameters.

In this hierarchical model, constraints on the posterior density over model parameters are provided by the level above. In variational Bayesian inference, the approximate posterior over the second level parameters is obtained by optimising its sufficient statistics (i.e., mean and covariance) with respect to a (second level) free energy:

3 $$\begin{aligned} \overset{\lower0.5em\hbox{$\smash{\scriptscriptstyle\frown}$}}{q}^{(1)*} &= \arg \max_{{\overset{\lower0.5em\hbox{$\smash{\scriptscriptstyle\frown}$}}{q}^{(1)} }} F^{(1)} (\overset{\lower0.5em\hbox{$\smash{\scriptscriptstyle\frown}$}}{p}_{F} ,\overset{\lower0.5em\hbox{$\smash{\scriptscriptstyle\frown}$}}{q}^{(1)} ) \\ \overset{\lower0.5em\hbox{$\smash{\scriptscriptstyle\frown}$}}{q}^{(2)*} &= \arg \max_{{\overset{\lower0.5em\hbox{$\smash{\scriptscriptstyle\frown}$}}{q}^{(2)} }} F^{(2)} (\overset{\lower0.5em\hbox{$\smash{\scriptscriptstyle\frown}$}}{p}^{(2)} ,\overset{\lower0.5em\hbox{$\smash{\scriptscriptstyle\frown}$}}{q}^{(2)} ,\overset{\lower0.5em\hbox{$\smash{\scriptscriptstyle\frown}$}}{q}^{(1)*} ) \\ \\ F^{(1)} (\overset{\lower0.5em\hbox{$\smash{\scriptscriptstyle\frown}$}}{p}_{R} ,\overset{\lower0.5em\hbox{$\smash{\scriptscriptstyle\frown}$}}{q}^{(1)} ) &= E_{{q^{(1)} }} [\ln p(y|\theta^{(1)} ,m)] - D_{KL} [q(\theta^{(1)} |\overset{\lower0.5em\hbox{$\smash{\scriptscriptstyle\frown}$}}{q}^{(1)} )||p(\theta^{(1)} |\overset{\lower0.5em\hbox{$\smash{\scriptscriptstyle\frown}$}}{p}_{R} )] \\ F^{(2)} (\overset{\lower0.5em\hbox{$\smash{\scriptscriptstyle\frown}$}}{p}^{(2)} ,\overset{\lower0.5em\hbox{$\smash{\scriptscriptstyle\frown}$}}{q}^{(2)} ,\overset{\lower0.5em\hbox{$\smash{\scriptscriptstyle\frown}$}}{q}^{(1)*} ) &= E_{{q^{(2)} }} [F^{(1)} (\overset{\lower0.5em\hbox{$\smash{\scriptscriptstyle\frown}$}}{p}_{R} ,\overset{\lower0.5em\hbox{$\smash{\scriptscriptstyle\frown}$}}{q}^{(1)} )] - D_{KL} [q(\theta^{(2)} |\overset{\lower0.5em\hbox{$\smash{\scriptscriptstyle\frown}$}}{q}^{(2)} )||p(\theta^{(2)} |\overset{\lower0.5em\hbox{$\smash{\scriptscriptstyle\frown}$}}{p}^{(2)} )] \\ \\ \overset{\lower0.5em\hbox{$\smash{\scriptscriptstyle\frown}$}}{p}_{R} &= (\varGamma (\theta^{(2)} ),\varSigma (\theta^{(2)} )) \\ \end{aligned}$$

The key thing about this free energy is that it can be evaluated (using BMR) without optimising the first level posterior. This means the second level parameters (e.g., group means) can be optimised or estimated, for any given model of priors, without reinventing the model at the first level. Technically, the inversion of the hierarchical or empirical Bayesian model only requires the posterior density from the inversion of each subject’s DCM. In short, the use of BMR allows one to make inferences at the group level without having to re-estimate subject-specific parameters; see (Friston et al. 2015, 2016) for details and a study of robustness of this scheme—and (Litvak et al. in press) for a reproducibility study using independent data under formally distinct models. Finally, after obtaining optimized second level estimates, these can be used as empirical priors to recursively optimize densities over parameters at the first level. The latter approach is not necessary but can finesse the local minima problem inherent in nonlinear (dynamic causal) modelling at the first level and allows one to estimate subject—or trial—specific parameters when a subset of subjects (or trials) provide more informative data than others (e.g. because of differences in lead fields), see (Friston et al. 2016).

**Fig. 1:**
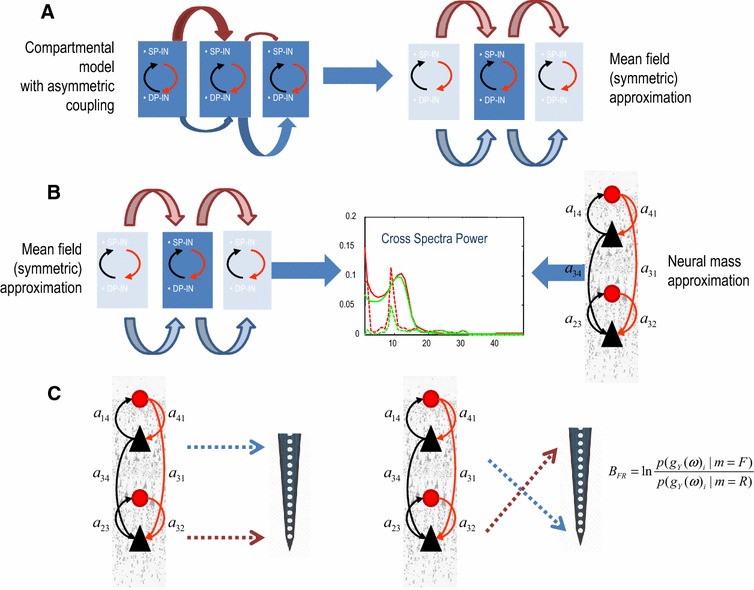
Schematic of the validation steps. **a** We first establish the functional equivalence between the model of Jones et al. (2007) and its symmetric variant. Here, *horizontal arrows* of different widths in the left panel denote asymmetric connectivities and delays between mini-columns—depicted as *rectangles* containing superficial and deep pyramidal cells (SP and DP) and inhibitory interneurons (II). In the *right panel* a mean field reduction of the model (and the symmetry assumptions about lateral connections) reveals a setup similar to that adopted in neural mass models. **b** We then establish the construct validity of the corresponding mass model in relation to mean field model above. This is achieved by fitting the model to synthetic data obtained from its compartmental homologue. **c** Finally, we show how this model can distinguish between superficial and deep responses obtained with laminar probes. We exploit Bayesian model selection and compute the relative log-evidence for plausible (*left*) and implausible (*right*) experimental setups, where the probes of laminar sensors are placed in the correct and inverted locations, see below

The second application of (approximate) Bayesian inference considered below uses posterior means obtained from compartmental (conductance based) models (Jones et al. 2007; Mainen and Sejnowski 1996; Prinz et al. 2003; Traub et al. 1991) as empirical priors. We first obtain simulated responses from a compartmental model that has been previously shown to faithfully represent the cortical microarchitecture—and has been used to model MEG responses during a tactile stimulation paradigm (Bush and Sejnowski 1993; Jones et al. 2007). We then use these simulated data to optimize the mean-field (lumped) parameters of a homologous neural mass model. The resulting parameters provide prior constraints on neural mass models that can be used for subsequent dynamic causal modelling of empirical responses. This approach ensures the neural mass model has construct validity, in relation to more detailed (compartmental) models of cortical microcircuitry; see Fig. 1 for a summary of this approach.

Our aim, in this second study, is to make inferences using data collected with laminar probes (see below). The first level generative model makes predictions of layer specific responses, where we eschew the difficult problem of inverting detailed (compartmental) models (due model complexity and conditional dependencies) by using simulated data obtained with a microscopic (compartmental) model $m_{CM}$ to inform a (mean field) neural mass model $m_{MF}$ of empirical data. In other words, we consider the joint optimization of compartmental and neural mass models, assuming they are homologous (i.e., they explain the same underlying cortical function and structure, see Pinotsis et al. under review, for more details). Effectively, we use the conditional densities obtained after fitting simulated data as empirical priors for subsequent analyses of empirical data. In what follows, we illustrate this approach after first describing a study of individual differences in gamma oscillations.

## Neural Models and Their Inversion with Variational Bayes

**Fig. 2:**
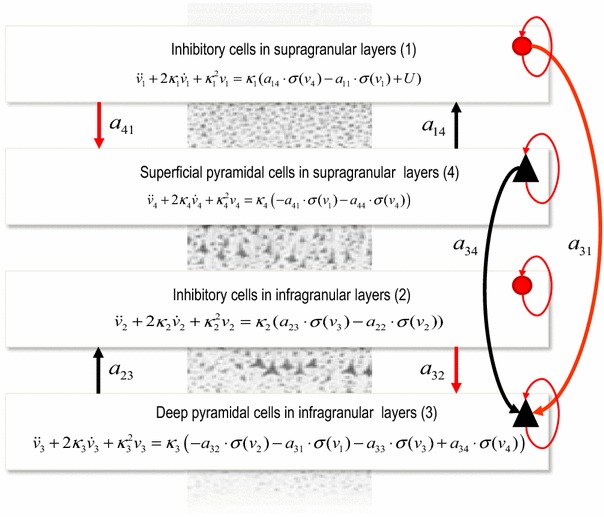
The Bush and Sejnowski mass model. This *figure* shows the evolution equations that specify a neural mass of a single source. This model contains four populations occupying different cortical layers: the pyramidal cell population of the Jansen and Rit model is here split into two subpopulations allowing a separation of the sources of forward and backward connections in cortical hierarchies. Firing rates within each sub-population provide inputs to other populations and subsequent convolution of presynaptic activity generates postsynaptic depolarization. We treat the activity in superficial and deep populations as separate predictors—as opposed to common neural mass model applications that use weighted mixtures of activity from all subpopulations. Excitatory connections are in *black* and inhibitory connections are in *red*

Neural models describe brain activity at different scales, ranging from single cells to whole brain networks. In this review, we consider both population and compartmental models. In the first application using Bayesian model reduction, we consider a neural field model with a canonical cortical microcircuitry following (Bastos et al. 2012; Pinotsis et al. 2014). In the second application—that focuses on the analysis of interlaminar data—we consider a compartmental model that follows the microcircuitry introduced in (Bush and Sejnowski 1993) and its neural mass counterpart introduced in (Pinotsis et al. under review), see Fig. 2.

Population models come in different flavours, for a review see (Deco et al. 2008; Moran et al. 2015) with some cardinal distinctions; namely the distinction between *convolution* and *conductance* dynamics, the distinction between *neural mass* and *mean field* formulations and the distinction between *point sources* and *neural field* models. The first distinction pertains to the dynamics or equations of motion within a single population. Convolution models formulate synaptic dynamics in terms of a (linear) convolution operator; whereas conductance based models consider the (non-linear) coupling between conductance and voltage. The second distinction is between the behaviour of a neuronal population or ensemble of neurons—as described with their mean or a *point probability mass* over state space. This contrasts with mean field approaches that model the ensemble density, where different ensemble densities are coupled through their expectations and covariances; in other words, these models include a nonlinearity that follows from the interaction between first and second order moments. This extra realism allows them to reproduce faster population dynamics; for example, somatosensory evoked potentials (Marreiros et al. 2010; Pinotsis et al. 2013b). Finally, there is a distinction between models of populations as point sources (c.f., equivalent current dipoles) and models that have an explicit spatial domain over (cortical) manifolds that call on neural fields. Neural field models are defined in terms of (integro-) differential equations that describe cortical dynamics in terms of (spatially) distributed sources sending afferent connections, conduction delays and lumped synaptic time constants (Pinotsis and Friston 2014b). These equations prescribe the activity in neuronal populations occupying bounded manifolds (patches) in different layers that lie beneath the cortical surface. In summary, field and mass models offer a coarse-grained description of spatiotemporal dynamics of brain sources in terms of smooth (analytic) connectivity matrices that also depend on time (and perhaps space).

Compartmental models on the other hand, operate at the single cell level. They yield precise descriptions of the anatomy, morphology and biophysical properties of the neurons that constitute populations. These models provide detailed descriptions of intracellular (longitudinal) currents within the long apical dendrites of synchronized cortical pyramidal cells, see e.g. (Bazhenov et al. 2002; Einevoll 2014; Krupa et al. 2008; Lindén et al. 2010; Ramirez-Villegas et al. 2015; Roth and Häusser 2001; Santaniello et al. 2015). These models embody the laminar structure of a cortical column and can characterize the cellular and circuit level processes that are measured with multielectrode arrays, MEG or electrocorticography. They provide characterizations of neuronal morphology and how neurons are grouped together to form spatially extended networks with well-behaved intrinsic (inter-and intra-laminar) connectivity. In the analysis of microelectrode data below, we employ a compartmental model that was originally used to explain somatosensory evoked responses measured with MEG during a tactile stimulation paradigm (Jones et al. 2007), and its neural mass analogue. Having specified the particular generative or forward model of observed that physiological responses the next step is to estimate the evidence and parameters of competing models; usually using dynamic causal modelling (DCM).

DCM offers a framework for the inversion of state space models using a Variational Bayesian algorithm known as Variational Laplace. This is based on the optimization of a cost function called variational Free Energy, ${\mathcal{F}}$. This provides a bound on the model log-evidence that—under Gaussian assumptions about the posterior density and random effects—acquires a simple form: see (Friston et al. 2007) for details. A standard model inversion corresponds to the case when ${\mathcal{F}}$ is given by Eq. (3), while the empirical Bayesian approach used here considers the case where ${\mathcal{F}}$ is defined at the first and second level on a hierarchical model (within and between subject respectively)—and is optimized with respect to first and second level posteriors (Eq. 3). Crucially, this optimization is computationally efficient, because the second level free energy receives a contribution from the first level that can be computed easily for any (reduced) priors, given the (pre-computed) posterior under full priors: see Friston et al. (2015, 2016) and subsequent applications for more details.

Compartmental models usually include a large number of parameters that renders the (ill-posed) inverse problem of mapping responses to laminar-specific neuronal sources quite hard. This mapping has been addressed using methods like current source density (Freeman and Nicholson 1975; Koo et al. 2015; Mitzdorf and Singer 1977; Sakamoto et al. 2015) and more recently Laminar population analysis (Einevoll et al. 2007; Ness et al. 2015). We show below that this problem can be bypassed using an alternative approach. This approach uses Bayesian model comparison and predictions from a compartmental model to establish a formal equivalence with a population model. After the this equivalence or construct validity has been established, variational Bayes can be used to invert the population model as described above. Bayesian model selection is based on the (variational free energy approximation to the) relative log-evidence of competing models (Bayes factor):

4 $$B_{ij} = \ln \frac{{p(y|m_{i} )}}{{p(y|m_{j} )}}$$

If $B_{ij} > 3$, we can say that model *m*_*i*_ is better than *m*_*j*_—or more exactly, there is strong evidence for the i-th model relative to the *j*-th model.

## Explaining Intersubject Variability in Gamma Responses Using Neural Fields

Neural rhythms are thought to reflect summed activity from excitatory and inhibitory pools of neurons under various input stimuli and in several cortical sources, e.g. (Hauck et al. 2007; Xing et al. 2009). We show below that by analysing such responses with neural field models and PEB, we can understand which mechanisms (manifested in the dynamics of coupled populations and lateral connections) are important for explaining individual differences in local gamma responses (or other phenotypes). In the following, we consider a particular form of Eq. (1) where $\varGamma_{i} (\theta^{(1)} )$ is a likelihood model that produces observed (cross spectral) responses at sensors *l* and *m* (Pinotsis et al. 2014):

5 $$\begin{aligned} \varGamma_{i} (\theta^{(1)} ) &= \varGamma_{i} (\theta^{(1)} ,\omega ) = \overset{\lower0.5em\hbox{$\smash{\scriptscriptstyle\frown}$}}{g}_{lm} (\omega ) + g_{n} (\omega ) + \varepsilon^{(1)} \\ \overset{\lower0.5em\hbox{$\smash{\scriptscriptstyle\frown}$}}{g}_{lm} (\omega ) &= \sum\nolimits_{k} {T_{l} (k,\omega )g_{u} (k,\omega )T_{m} (k,\omega )^{\dag } } \\ \\ T_{r} (k,\omega ) &= L_{r} (k,\varphi )Q \cdot T(k,\omega ,\theta^{(1)} ) \\ g_{n} (\omega ) &= \alpha_{n} + {{\beta_{n} } \mathord{\left/ {\vphantom {{\beta_{n} } \omega }} \right. \kern-0pt} \omega } \\ g_{u} (\omega ) &= \alpha_{u} + {{\beta_{u} } \mathord{\left/ {\vphantom {{\beta_{u} } \omega }} \right. \kern-0pt} \omega } \\ \\ Re(\varepsilon^{(1)} )&\sim {\mathcal{N}}(0,\varSigma (\omega ,\lambda )) \, \text{Im} (\varepsilon^{(1)} )\sim {\mathcal{N}}(0,\varSigma (\omega ,\lambda )) \\ \end{aligned}$$

Here, $L_{r} (k,\varphi )$ is the Fourier transform of the lead field of the *q*-th sensor, $\dag$ denotes the conjugate transpose matrix and $Q = [q_{1} ,q_{2} ,q_{3} ,q_{4} ]$ is a vector of coefficients that weights the contributions of each neuronal population to the observed MEG signal. Here, $g_{u} (\omega )$ is a spatiotemporal representation of fluctuations or inputs driving induced responses, which we assume to be a mixture of white and pink temporal components. These contributions are based on anatomical properties and the lead field configuration of each population (e.g. inhibitory neurons do not generate a large dipole), where each electrode or sensor has its own sensitivity profile, reflecting the topographic structure of the underlying cortical source.

Equation (5) describes the predicted cross spectra as a function of the power of underlying neuronal fluctuations $g_{u} (\omega )$ and transfer functions $T(k,\omega ,\theta^{(1)} )$ that depend upon model parameters at the first or within-subject level: $\theta^{(1)}$, see Table 1. For the explicit form of the transfer functions $T(k,\omega ,\theta^{(1)} )$ we refer the interested reader to (Pinotsis et al. 2014).

**Table 1:** Neural field model parameters

Parameter	Physiological interpretation	Prior mean
$\kappa_{1} ,\kappa_{2} ,\kappa_{3} ,\kappa_{4}$	Postsynaptic rate constants	1/2, 1/35, 1/35, 1/2 (ms^−1^)
$\alpha_{11} ,\alpha_{14} ,\alpha_{12}$ $\alpha_{22} ,\alpha_{21} ,\alpha_{23} ,\alpha_{33}$ $\alpha_{41} ,\alpha_{32} ,\alpha_{44}$	Amplitude of intrinsic connectivity kernels (×10^3^)	108, 45, 1.8 9, 162, 18, 45 (a.u) 36, 18, 9
$c_{ab}$	Spatial decay of connectivity kernels	$$\left\{ {\begin{array}{*{20}c} {0.6} & {{\text{a}} \ne b} \\ 2 & {a = b} \\ \end{array} } \right.$$ (mm^−1^)
$r,\eta$	Parameters of the postsynaptic firing rate function	.54, 0 (mV)
*s*	Conduction speed	.3 m/s^3^
$\phi$ $q_{1} ,q_{2} ,q_{3} ,q_{4}$	Dispersion of the lead field Neuronal contribution weights	$\sqrt 2 /16$ (mm) .2, 0, .2, .6
$a_{u} ,a_{n}$	Exogenous white input, channel-specific white noise (log–scale)	0, 0
$\beta_{u} ,\beta_{n}$	Exogenous pink input, channel-specific pink input (log–scale)	0, 0

Below we use the likelihood model given by Eq. (5) and PEB to study intersubject variability in (stimulus-locked) oscillations recorded with MEG during a visual perception paradigm (Perry et al. 2013). Technical details of this analysis can be found in (Pinotsis et al. to appear). Here, our focus is on explaining these results from the vantage point of hierarchical Bayesian inference (see above). We used cross spectral densities as data features that were taken from observed responses, while the subject was looking at stationary, vertically oriented bars. These spectral responses showed sustained activity in the 30–80 Hz range that varied across individuals with stimulus size: these responses either showed an approximately linear (monotonic) increase in the gamma-band response or a saturating response with increasing size, akin to surround suppression. So what are the key mechanisms that could explain these individual differences?

**Fig. 3:**
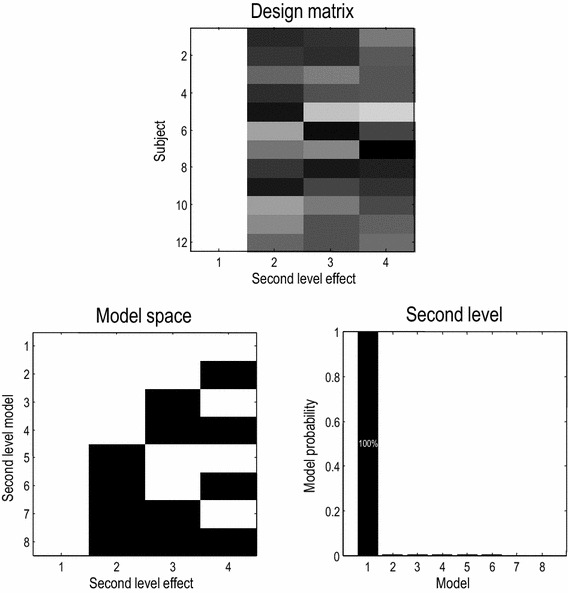
*Above* design matrix containing the between subject effects; these include a constant term and three parametric variables based upon electrophysiological characterisations of each subject. *Left* model space comprising second level effects encoded by the design matrix. *Right* posterior probability over models shows all three between subject effects are necessary to explain between subject gamma response variability

As intimated earlier, inferences and Bayesian model comparison at the group level necessitate the use of hierarchical models. In other words, $\varGamma (\theta^{(2)} ) = (X \otimes W)\beta$, where *X* and *W* are design matrices describing group and within subject effects respectively. In our application, we assume (for simplicity), $W = I$ and consider three proxies to describe phenotypic variations between subjects; namely, the change in amplitude of gamma responses with increasing stimulus size, the peak frequency over all stimuli and the amplitude of gamma responses averaged over stimuli (Perry et al. 2013). These proxies or phenotypes enter the design matrix *X*, creating the model space depicted in Fig. 3. First, we fit the (first level) model to individual subject data as in standard DCM approach. Then, we use Bayesian model reduction to invert the hierarchical model (2). The Kronecker tensor product with the identity matrix $X \otimes I$ means that we have a second level parameter for every second level (phenotypic) variable and every first level (connectivity) parameter. This means one can identify the combination of connectivity parameters and phenotypic variables that best explains intersubject variability. This model space corresponds to all combinations of between subject effects. Having defined the hierarchical model we can now establish the significance of any group effects using Bayesian model reduction over (second level) models; see Fig. 3.

**Fig. 4:**
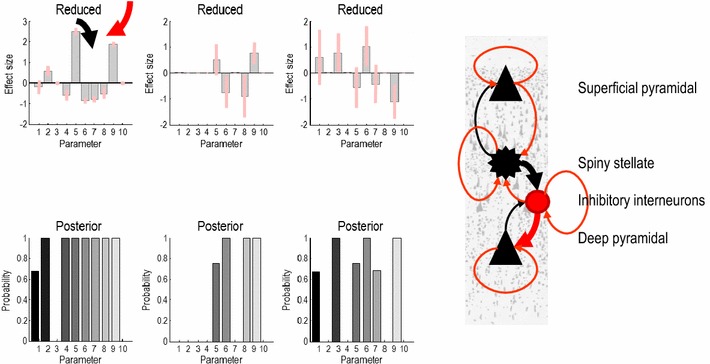
Posterior estimates obtained using BMR. Second level effects comprised differences in gamma responses with stimulus size and associated gamma peak frequency. Posterior means are in *grey* and 90 % confidence intervals are depicted in *red*. Individual differences in spectral responses seem to implicate connections to and from inhibitory interneurons (intrinsic connections five and nine are highlighted in *thick lines* in the insert on the *right*: inhibitory cells and connections are shown in *red*, while excitatory populations and connections are shown in *black*). Model posteriors for models with and without each second level parameter are shown separately for the constant term or group mean (*bottom left panel*) and group effects (*bottom middle* and *right panels*)

Having established the importance of the (between subject) explanatory variables, we can now focus on the main question; in particular, which factors mediate intersubject variability in gamma band responses. To address this question, we performed an exhaustive search over combinations of second level parameters, that is, the 30 parameters describing the effects of the three explanatory variables on the ten intrinsic connections of our neural field model. Using BMR, we scored every combination of parameters and identified key connections explaining intersubject variability in gamma responses. Bayesian model averages of these parameters are shown in Fig. 4: interestingly, individual differences can be explained by connections to and from inhibitory interneurons within a local source in the visual cortex. This speaks to crucial rule of fast inhibitory interneurons for the genesis of gamma rhythms—often referred to as the ING hypothesis (Lytton and Sejnowski 1991; Whittington et al. 2000). This example shows how one can obtain mechanistic explanations for intersubject variability in the peak gamma frequency, observed during visually induced oscillations and follows a similar study that asked whether this variability can be attributed to cortical structure or function (Pinotsis et al. 2013a). In contrast to the earlier paper—that used a summary statistic approach—the current study used a purely probabilistic approach based on empirical Bayesian models.

In short, this example shows how non-invasive (macroscopic) data can be used to make inferences at a microcircuitry level. In this instance, we have taken the opportunity to highlight inferences in the setting of hierarchical or empirical Bayesian models that accommodate intersubject variability. Our conclusion is that intersubject variability in visually induced gamma responses is best explained by differences in the intrinsic (laminar) connectivity to and from inhibitory interneurons.

## Modelling Layer-Specific Activity Using Neural Mass Models

We now turn to our second example that involves laminar responses and a slightly different neural circuitry that follows the architecture used in (Bush and Sejnowski 1993). We consider two versions of Eqs. (1); these correspond to a compartmental and a neural mass model respectively

6 $$\begin{aligned} \Gamma_{CM} (\theta^{(1)} ) &= \sum\limits_{m} {A_{m} l_{m} L_{im} J_{m} (t)}\\ J_{m} &= - \frac{{\pi A_{m}^{2} }}{{\rho_{m} }}\nabla_{x} v_{m} \\ \dot{v}_{m} &= h(v_{m} ,U,\theta ) = c_{m}^{ - 1} [1/(2A_{m} \rho_{m} )\nabla_{x} (A_{m}^{2} \nabla_{x} v_{m} ) + U] \\ \Gamma_{NM} (\theta^{(1)} ) &= \sum\limits_{q} {L_{q} (\varphi )} \ddot{V} _{q} (t), \, q = 1,\ldots,4 \\ {\ddot{V} }_{q} = f_{2} (V_{m} ,U,\theta^{(1)} ) &= - 2\lambda_{q} \ddot{V} _{q} - \lambda_{q}^{2} {\ddot{V} }_{q} + \lambda_{q} f_{q} (v_{q} ,U,\theta )\\ f_{q} (v_{q} ,U,\theta ) &= \left\{ {\begin{array}{*{20}c} {a_{14} \cdot \sigma (v_{4} ) - a_{11} \cdot \sigma (v_{1} ) + U, \, q = 1} \\ {a_{23} \cdot \sigma (v_{3} ) - a_{22} \cdot \sigma (v_{2} ), \, q = 2} \\ { - a_{32} \cdot \sigma (v_{2} ) - a_{31} \cdot \sigma (v_{1} ) - a_{33} \cdot \sigma (v_{3} ) + a_{34} \cdot \sigma (v_{4} ), \, q = 3} \\ { - a_{41} \cdot \sigma (v_{1} ) - a_{44} \cdot \sigma (v_{4} ), \, q = 4} \\ \end{array} } \right.\\ \end{aligned}$$

The first of the above models is a well-known conductance based (microscopic) model (Bush and Sejnowski 1993). In this model, neurons and their constituent parts (axonal arbours, soma etc.) are considered as cylindrical conductors (segments) and transmembrane potentials are given by aggregates of Ohmic currents. These currents flow across the compartment, forming an RC circuit and obey Kirchhoff’s law. $L_{im}$ are lead field coefficients for each compartment and sensor, $A_{m} ,l_{m}$ are the cross-sectional area and the length of compartment *m* (projected in a direction perpendicular to apical dendrites). $\rho_{m}$, $c_{m}^{{}}$ are the axial resistivity and membrane capacitance and $J_{m} (t)$ is the longitudinal current density. This model yields detailed descriptions of intracellular longitudinal currents—within the long apical dendrites of synchronized cortical pyramidal cells—that follow from cable theory. Neuronal populations are modelled as spatially organised networks with the soma of principal cells in supragranular and infragranular layers. This model captures the laminar structure of cortical columns and can characterize the cellular and circuit level processes that are measured with multi-electrode arrays or MEG. It also provides a model of neuronal morphology and how neurons are grouped together to form spatially extended networks, with precise connectivity.

The second model considered above is the neural mass variant of the Bush and Sejnowski 1993) model, see Fig. 2. The crucial difference between the two models in Eq. (6) is that the latter operates at the mesoscale and cannot describe microscopic effects like dendritic delays or back propagation. However, by fitting responses generated by its homologous microscopic model (using DCM), we obtain a prior distribution of neural mass model parameters that can faithfully explain responses recorded with laminar probes, see also Pinotsis et al. under review. In other words, we can establish a mapping between detailed compartmental models based upon conductances and simpler neural mass models based upon (implicit) synaptic convolutions. This mapping uses exactly the same inference machinery used to analyse empirical data but, in this instance, we are fitting neural mass models to responses that are generated by detailed compartmental models.

## A Working Memory Task and Experimental Data

Recordings were obtained from a monkey performing a memory guided saccade task. The monkey was trained to fixate on a central white dot during the 250 ms presentation of a red dot (cue) in the periphery of the animal's vision. This cue was presented at one of six potential locations evenly spread on an annulus 10° from the fixation point. After the cue, dots appeared at all of the six locations while the monkey maintained central fixation over a two second memory delay. Then, the central fixation dot turned purple and the peripheral stimuli disappeared. This told the monkey to make a direct saccade to the remembered location of the red cue dot to receive a juice reward. We recorded local field potentials from a 24 channel multi-contact laminar electrode implanted within prefrontal cortex.

**Fig. 5:**
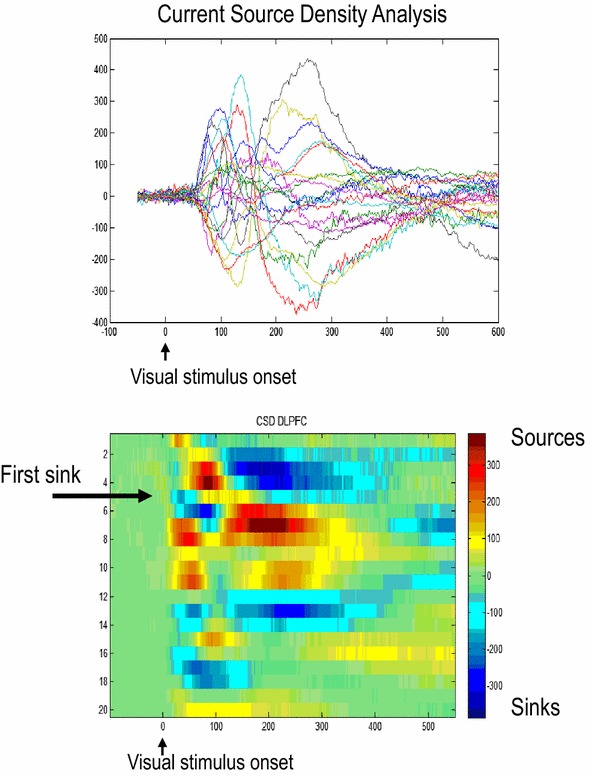
Current source density channels (*top*) and profile across channels (*bottom*). We find that the first active sink corresponds to unipolar channel 7. This enables us to distinguish contacts that measure responses from distinct cortical layers, that is superficial (contacts 3–4) and deep (contacts 9–10) populations

To help locate the electrode relative to the cortical layers, we also trained the monkey to maintain fixation while a white disk with a radius of 11 visual degrees was repeatedly flashed for 50 ms intervals. A current source density analysis of the visually evoked potentials during this task permitted the identification of the cortical lamina surrounding the electrode. (Godlove et al. 2014). An example of this type of analysis is shown in Fig. 5. This figure illustrates peristimulus time responses of current source density channels (top) and the corresponding current source density depth variations (bottom) from a laminar probe placed in the prefrontal cortex over all cortical layers (Mitzdorf and Singer 1978). This analysis reveals current sinks and sources and allowed us to identify the first active sink, which corresponds to middle (granular) layers of cortex. Thus, we can localize each of the 24 channels of the laminar electrode in relation to the granular layer—and determine whether they lie in the superficial as opposed to deep layers. In the final part of this review, we use this analysis to establish the validity of our neural mass model above and show that it can successfully explain layer specific responses.

## Dynamic Causal Modelling of Laminar Probe Data

After equiping the mass model of Fig. 2 with with priors that are consistent with compartmental models (see above), we inverted the empirical responses induced during the delay period in the working memory task. We then used Bayesian model comparison to test whether the model could successfully identify the layer (superficial vs deep) from which we recorded responses.

**Fig. 6:**
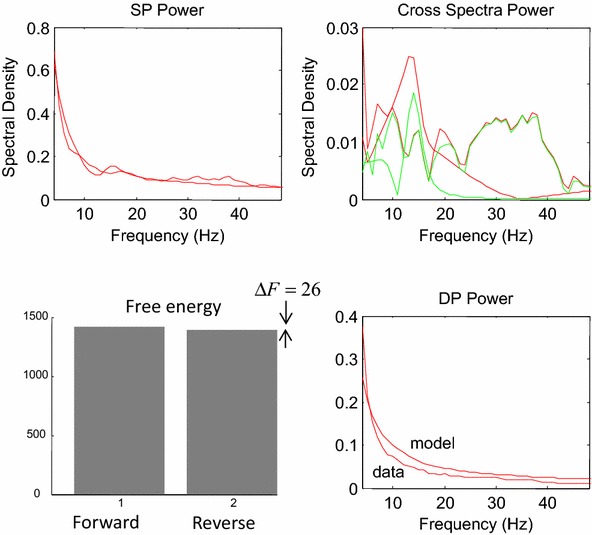
Spectral responses and model fits during the delay period from pairs of superficial and deep contacts across the laminar probe. These fits used bipolar data from the delay period, averaged across all conditions, calculated using Hanning tapers. Model predictions are in *red* and empirical (spectral) data features in *blue*. The *inset* shows a log-evidence difference for models with the correct and incorrect designation of laminar depth (two superficial and deep pyramidal cell populations). This relative evidence shows that the model can correctly distinguish between responses originating from different layers

We find that although this model is formulated at a mesoscopic scale, it could indeed distinguish between activity arising from different layers, see Fig. 6. For example, Bayesian model comparison revealed a relative log-evidence of 26 when we swapped supercial and deep recordings. Generally, a relative log-evidence of three or more can be taken as strong evidence for one model over another (Kass and Raftery 1995). The remarkable thing about this result is that the neural mass model has no explicit notion of space or laminar depth. In other words, the distinction between superficial and deep populations rests purely on their connectivity and synaptic time constants, without any explicit reference to their spatial deployment across multiple cortical layers. The (correct) mapping between superficial and deep populations to their laminar depth endorses or validates the prior constraints on their respective (synaptic) parameters—that can generate complicated mixtures of spectral responses.

This sort of validation is potentially important as laminar probes offer unbiased estimates of laminar specific activity—and the hierarchical architecture of extrinsic (between-source) connections rests primarily on laminar specific connectivity. Laminar probes are therefore an exciting technique that allows us to measure brain responses at an unprecedented resolution. When combined with dynamic causal modelling, these responses could be used to address several important questions that we review in the Conclusions below.

## Conclusion

We have reviewed two recent advances in hierarchical or empirical Bayesian modelling that enable us to deal with the ill-posed (inverse) problem of source reconstruction and disclose processes that generate neural rhythms—and mediate the propagation of information within and between cortical sources. In the first illustration, we showed that non-invasive recordings contain rich spatial information, despite the low resolution of M/EEG; while in the second, we attempted to reconcile models operating at the microscopic and mesoscopic scale—and show that DCM can correctly assign superficial and deep cortical dynamics to laminar-specific responses. This intralaminar DCM affords the same computational efficiency and advantages as all other models in DCM, but can exploit microscopic (laminar-specific) data that embody effects like antidromic currents and back-propagation.

Dynamic causal modelling of electrophysiological responses obtained with laminar probes is in a position to address several neurobiological questions: one of the key reasons to use laminar probes is that they can provide direct evidence that distinct cortical layers are involved in particular oscillations and computations. Previous studies of visual cortex suggest that oscillatory activity in the gamma and alpha bands are segregated by layers. Neurons in deep layers (layers 5 and 6) show spike-field coherence in the alpha band while superficial layer (layers 2 and 3) neurons show spike-field coherence in the gamma band (Buffalo et al. 2011). A question of outstanding importance is whether this laminar segregation is preserved in prefrontal cortex, which is involved in top-down control of sensory cortex (Miller and Cohen 2001).

Superficial and deep cortical layers also tend to have distinct cortical targets. For example, superficial-layer neurons form the strongest source of cortico-cortical feedforward projections, while deep-layer neurons contribute predominately to cortico-cortical feedback (Markov et al. 2013). Recently, it was shown that the laminar connectivity pattern of a particular inter-areal (extrinsic) connection predicts how inter-areal oscillatory activity is coordinated between the areas: when a given connection is dominated by superficial-layer projection neurons (characteristic of feedforward connectivity), gamma and theta oscillations predominate. On the other hand, when a reciprocal connection is dominated by deep-layer projection neurons (characteristic of feedback connectivity), beta oscillations appear to mediate neuronal communication (Bastos et al. 2015). These results suggest that the precise laminar pattern of extrinsic connectivity profoundly shapes inter-areal communication, and the frequencies over which it occurs.

Therefore, it appears that the functional role of oscillations is shaped both by cortical layer and inter-areal connection types, as reviewed above. This provides an important motivation for using multi-laminar probes to examine cortical activity during cognitive tasks. An equally important motivation, from our perspective, is to interrogate the canonical microcircuit hypothesis, which predicts that neurons in distinct cortical layers contribute to distinct computations (Bastos et al. 2012; Friston and Kiebel 2009; Friston et al. 2015). In particular, it has been hypothesized that superficial layer neurons can encode prediction error, while deep layer neurons encode expectations that are used to generate descending (feedback) predictions. The hierarchical message passing of prediction errors and predictions is thought to be a crucial part of predictive coding under the Bayesian brain hypothesis (Friston and Kiebel 2009; Rao and Ballard 1999; Summerfield et al. 2008). Therefore, multilaminar data may provide the critical test for these hypotheses: the modelling of these data could establish whether neuronal activities (spikes and LFPs) from different cortical layers are indeed involved in distinct computations implied by predictive coding. In parallel, these data can be used to inform and nuance laminar-resolved dynamic causal models of the sort we entertain here. In turn, more advanced models will provide more precise descriptions of laminar-resolved activity, allowing more mechanistic questions to be asked about the role of specific neuronal populations, intrinsic connectivity and their neuromodulators in cognition.
